# Vertebrate odorant binding proteins as antimicrobial humoral components of innate immunity for pathogenic microorganisms

**DOI:** 10.1371/journal.pone.0213545

**Published:** 2019-03-22

**Authors:** Federica Bianchi, Sara Flisi, Maria Careri, Nicolò Riboni, Silvia Resimini, Andrea Sala, Virna Conti, Monica Mattarozzi, Simone Taddei, Costanza Spadini, Giuseppina Basini, Stefano Grolli, Clotilde Silvia Cabassi, Roberto Ramoni

**Affiliations:** 1 Department of Chemistry, Life Sciences, and Environmental Sustainability, University of Parma, Parma, Italy; 2 Department of Veterinary Sciences, University of Parma, Parma, Italy; City University of Hong Kong, HONG KONG

## Abstract

The bacterium *Pseudomonas aeruginosa* (PA) and the yeast *Candida albicans* (CA) are pathogens that cohabit the mucosa of the respiratory tracts of animals and humans. Their virulence is largely determined by chemical communication driven by quorum sensing systems (QS), and the cross perception of their quorum sensing molecules (QSM) can modulate the prevalence of one microorganism over the other. Aiming to investigate whether some of the protein components dissolved in the mucus layering the respiratory mucosa might interfere with virulence and cross-communication of these, and eventually other microorganisms, ligand binding assays were carried out to test the scavenging potential of the bovine and porcine forms of the Lipocalin odorant binding protein (OBP) for several QSMs (farnesol, and acylhomoserine lactones), and for pyocyanin, a toxin produced by PA. In addition, the direct antimicrobial activity of the OBPs was tested by time kill assay (TKA) against CA, PA and other bacteria and yeasts. The positivity of all the ligand binding assays and the antimicrobial activity determined for CA, and for some of the other microorganisms tested, let hypothesize that vertebrate OBPs might behave as humoral components of innate immunity, active against pathogenic bacteria and fungi. In addition, TKAs with mutants of bovine OBP with structural properties different from those of the native form, and with OBP forms tagged with histidines at the amino terminal, provided information about the mechanisms responsible of their antimicrobial activity and suggested possible applications of the OBPs as alternative or co-adjuvants to antibiotic therapeutic treatments.

## Introduction

The Gram-negative bacterium *Pseudomonas aeruginosa* (PA) and the yeast *Candida albicans* (CA), which are commonly found in mixed infections in numerous animal species and in humans, are examples of pathogenic microorganisms that can be fatal in both immunocompromised patients and in subjects affected by cystic fibrosis [1,2]. Their pathogenicity is markedly influenced by a complex network of ‘intra’ and ‘inter-specific’ chemical communication mechanisms belonging to the so-called quorum sensing systems (QS) [3,4]. Each QS is characterized by a signal compound, named quorum sensing molecule (QSM), that is produced by a synthase (I-protein), released into the environment and specifically recognized by a receptor (R-protein) of other microorganisms of the same species present in the same niche [4]. This allows the whole population of microorganisms to produce a unique common synchronized biosynthetic response only when the QSM concentration reaches a specific threshold. Several classes of compounds have been identified as quorum sensing molecules in different bacterial species, such as peptide-pheromones, N-acyl-homoserine lactones (AHLs), interconvertible furanones, hormones, quinolones and fatty acids [5]. AHLs are the most common and well-characterized gram-negative bacterial autoinducers: they can vary in acyl chain length from C4 to C18 and, depending on the bacterial species and the QS network involved, they can present unsaturation at the C-7 or C-8 position and/or oxidation at the 3 position [6].

In PA, QSs can regulate about 10% of the whole genome of the microorganism [7,8], thus indicating that QSMs can play relevant roles in the metabolism of this bacterium. Three hierarchically interconnected QSs have been identified in PA, namely LasI/LasR, RhlI/RhlR and PQS [9], playing LasI/LasR a ‘leading’ role on the others [10]. The first two QSs synthesize and respond to QSMs belonging to the same N-AHL chemical class, but with differences in the acyl group. More precisely, N-(3-oxododecanoil)-L-homoserine-lactone (oxo-C12AHL) is the QSM of LasI/LasR, whereas N-butyryl-L-homoserine-lactone (C4AHL) characterizes RhlI/RhlR. As for PQS, it is modulated by LasI/LasR and it is associated with 2-heptyl-3-hydroxy-4-quinolone, commonly released by PA through membranous vesicles. Similarly, the yeast CA produces QSMs that regulate major morphological and metabolic activities responsible of its virulence [11]. Among these, a pivotal role is played by the terpenoid farnesol, able to affect both the transformation from the mycelial to the yeast state, through the inhibition of the Ras-1 controlled pathway [12], and the resistance of CA to oxidative stress [13]. Moreover, the cross-perception of the QSMs released in the environment by PA and CA can affect the interactions between these microorganisms: the influence of oxo-C12AHL on the adherence of polymicrobial biofilms of CA [14] and the inhibition of *Pseudomonas aeruginosa* PQS exerted by farnesol [15] are two examples of these interactions.

The competition between microorganisms that cohabit in the same niche can also be tuned by quorum quenching (QQ) activities based on specific enzymes able to inactivate the QSMs of the competitors [16, 17]. Although mostly expressed by microorganisms, recently QQ enzymes were identified also in vertebrates [18], thus indicating that the host can adopt enzymatic QQ-based strategies to interfere with QS-driven synchronized virulence gene expression of the pathogen microorganisms. In addition to the enzyme-based inactivation, a further QQ strategy could be the QSMs removal operated by protein scavengers characterized by QSM-binding capacities.

Odorant binding proteins (OBP) are protein carriers for small hydrophobic molecules dissolved at millimolar levels in the mucus layering the epithelia of all the tracts of the respiratory apparatus of vertebrates [19,20]. OBPs belong to Lipocalins [21], a superfamily of structurally related protein expressed in bacteria, plants and animals that, despite low sequence homologies, share a common structural frame named Lipocalin folding, constituted by a beta barrel externally flanked by a carboxy-terminal alpha helix. The beta barrel comprises two thirds of the protein residues starting from the amino terminal (about 100 amino acids) and defines an internal cavity layered by non-polar amino acid residues, which represents the ligand binding site. The main peculiarity of the OBPs is the broad specificity of their ligand binding site, that can accommodate a large variety of moderately hydrophobic structurally unrelated organic compounds belonging to different chemical classes, whose common feature is a molecular mass ranging between 150 and 350 Da [22–25]. Previous literature data, dealing with the bovine and porcine forms of OBP (bOBP and pOBP, respectively), include as potential ligands of OBP both terpenoids, structurally related to farnesol, and molecules with physico-chemical features comparable to those of AHLs [26–28], thus suggesting that the OBP might exert *in-vivo* a scavenging based QQ activity against CA and PA.

As for the bovine and porcine forms of OBP, despite 42% sequence homology, a relevant difference in the three-dimensional structure is observed. In fact, while pOBP is a classical momomeric Lipocalin, the bovine form is a homodimer with domain swapping: each of the two Lipocalin folding units constituting the quaternary structure of the protein is formed by the beta-barrel of one subunit and the alpha helix of the other [29,30]. Even though the role of the OBPs has not yet been univocally defined, in the case of the respiratory apparatus it has been supposed that they can exert protection against oxidative stress by scavenging from the mucus highly reactive low molecular aldehydes and alken-aldehydes therein produced in consequence of peroxidation of membrane unsaturated fatty acids [23,31].

In the present study, the binding capability of both bOBP and pOBP toward farnesol and several AHLs, including oxo-C12AHL and C4AHL, were tested in order to evaluate the attitude of OBPs of operating *in vivo*, a scavenging-based QQ. The binding properties of the OBPs were also evaluated on pyocyanin, a toxin produced by PA under QS control [4,8,9 and 10], playing an important role in its pathogenicity, whose removal from the environment by scavenging might affect the virulence of the bacterium. To verify whether the OBPs can be effective as direct antimicrobial agents on living microorganisms, growth inhibition tests (time kill assay—TKA) were performed against CA, PA and other yeast and bacterial strains.

Due to the partial positivity of these tests, the contribution of the tertiary and quaternary structures on the efficacy of the antimicrobial activity of the proteins against CA was evaluated using the two functional deswapped monomeric mutants of bovine OBP, namely M3-bOBP (with the alpha helix freely movable in solution) [32] and GCC-bOBP (with a reconstructed monomeric Lipocalin folding) [33] characterized by the same ligand binding specificity and affinity with respect to the native dimeric form.

Moreover, further TKAs were realized against bacteria and fungi different than PA and CA, with the aim of investigating whether the OBPs can act as antimicrobial against a wide spectrum of pathogenic microorganisms.

Finally, with the aim of evaluating the possibility of a biotechnological applications of these proteins as antimicrobials, TKA tests were performed also with both bovine and porcine OBPs tagged with six histidine residues at the amino terminal [23], since the histidine content is considered a crucial element to provide histatines, natural peptides dissolved in saliva, with antifungal properties against CA [34].

## Materials and methods

The *Materials and Methods* are reported in details in Supporting information S1 Material and methods. Here are indicated the titles of the chapters of that section, and for some of them is reported a brief description of the procedures adopted.

### bOBP and pOBP biosynthesis and purification

The synthesis and purifications of all the OBP forms (both native and mutants) employed in the present study have been realized, with minor modifications, according to previously published procedures which are described in details it in S1 Material and methods.

### OBP ligand binding tests

The binding tests for farnesol, AHLs and pyocyanin are described in, S1 Material and methods.

Due to the equivalence of their binding properties [23], the ligand binding tests were realized with OBPs tagged with six histidine residues at the amino terminal that, with respect to the native forms, can be prepared at much higher amounts and purified by a single affinity chromatography step [22, 23 and 25]. As a positive control of the functionality of the of the OBPs, their binding capacities were first determined by direct titrations using the fluorescent ligand 1-amino-anthracene, according to previously published procedures [23].

Data from the literature report that the concentrations of farnesol, pyocyanin and AHLSs in different biological samples can range from nanomolar to micromolarar levels [35–40]. Nevertheless, since their biological activities are exerted at levels ranging from micromolar to submillimolar [9, 11, 40 and 41], we employed ligand binding assays that could show if the OBPs have the capability to scavenge molecules at these concentration levels.

On these basis, the dissociation constants (K_d_) of the complexes between the odorant binding-proteins and farnesol, whose structure and hydrophobicity is similar to that of most of the typical ligands of the OBPs, were determined by a competitive binding test at equilibrium with the fluorescent ligand 1-aminoanthracene [23], a method that allows to assess OBP-ligand interactions with K_d_ values in the micromolar range. Conversely, since the structures and/or polarity of AHLs and Pyocyanin, compared to farnesol, differ more from those of the typical OBP ligands, their binding was assessed following a procedure where the protein is more concentrated (sub-millimolar) than in the case of the competitive binding assay with 1-aminoanthracene (micromolar). In particular, the test was then performed by using filtering devices having pOBP and bOBP as retention elements [22], and by analyzing the eluted solutions *via* gas chromatography mass spectrometry (GC-MS) or liquid chromatography-tandem mass spectrometry (LC-MS/MS) analysis. This method is not suitable to determine the exact values of the dissociation constants, but in consequence of the high concentration of ligand binding sites, has the advantage that allows to detect the interactions with compounds that the OBPs bind with weaker affinities (with K_d_ values approximately higher than 10 micromolar) than 1-aminoanthracene. It must be underlined that, since sub-millimolar OBP concentrations are values comparable to those found in the mucus layering the epithelia of the respiratory mucosa [19], this binding assay could mimic, in terms of protein levels, the physiological condition of this biological fluid.

### Microbiological assays

Antimicrobial activity was assessed in function of time contact of the OBPs with different microorganisms, through time-kill assays as reported in S1 Material and methods.

## Results

### Vertebrate OBPs bind farnesol, AHLs and pyocyanin

**Fig 1:**
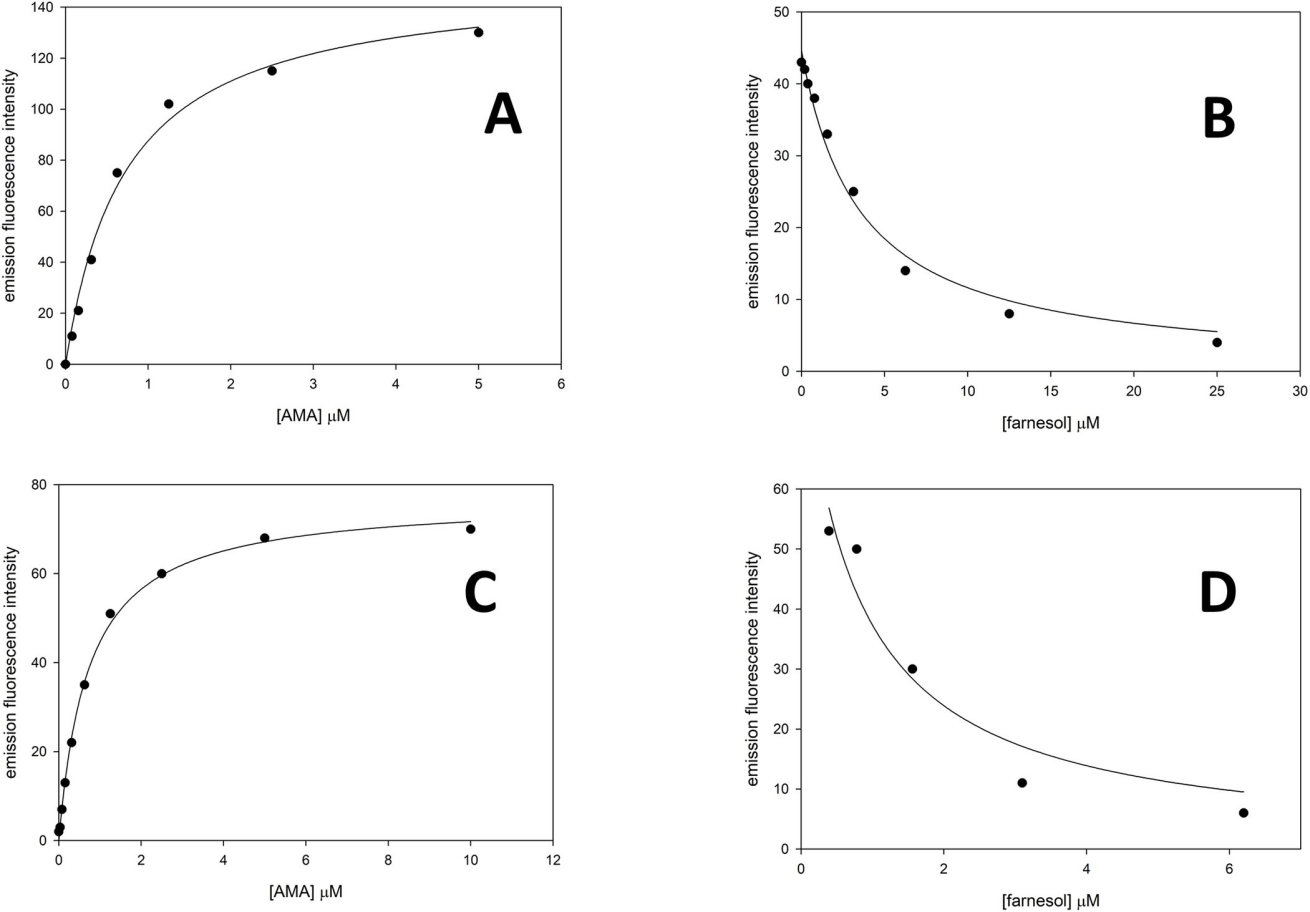
**Investigation of the binding of 1-aminoanthracene (A and C) and farnesol (B and D) to bovine (A) and porcine (B) OBP tagged with six histidine residues**. The graphs report either the direct binding (A and C), or the chasing of the OBP fluorescent ligand 1-aminoanthracene in response of increasing concentrations of farnesol (B and D). The amount of 1-aminoanthracene bound to OBP is expressed on the y axis as arbitrary units of emission fluorescence intensity. The four graphs report the results of single experiments. The curves were treated either as hyperbolic increases (A and C) or decays (B and D), with r values higher than 0.97. (The original data of the graphs are reported in S1 Available data).

The functionality of the bovine and porcine OBP was first assessed by testing their binding capacity for the fluorescent ligand 1-aminoanthracene. The hyperbolic titration curves (Fig 1A and 1C) gave K_d_ values of 0.72 and 0.77 μM respectively for the bovine and the porcine forms, that are values in agreement with those of functional preparations of the protein [22, 23, 24 and 25].

The affinity for farnesol was then evaluated by measuring the progressive chasing of AMA bound to bOBP and pOBP, in the presence of increasing concentrations of the ligand. As shown in Fig 1B and 1D, the competition curves could be treated as two hyperbolic decays with K_dapp_ (which is the concentration of farnesol that displaced 50% of the AMA bound to the OBP) values of 3.7 and 0.77 μM respectively for the bovine and the porcine form.

Since the calculated values of the dissociation constants (K_d_ = 0.4 and 2.3 μM for pOBP and bOBP, respectively) were in agreement with those determined for ligands that are typical for these kinds of protein [27,28], it can be supposed that both the OBP forms could effectively bind farnesol, with affinities in the micromolar range.

The binding capabilities of the two different forms of odorant binding protein were tested also for pyocyanin and several AHLs (C4AHL, C6AHL, C7AHL, oxo-C10AHL and oxo-C12AHL) by using filters filled in with 6-His tagged bOBP and pOBP, respectively, coupled at submillimolar levels to a Ni-NTA agarose resin. Both pyocyanin and AHL solutions were eluted through filters previously washed with phosphate buffer. Then, the filtered solutions were submitted to UV-Vis, GC-MS or LC-MS/MS analysis in order to quantitate the eluted ligands. Validation parameters of the proposed methods are reported in S1 Table (The original data are reported in S1 Available data).

**Fig 2:**
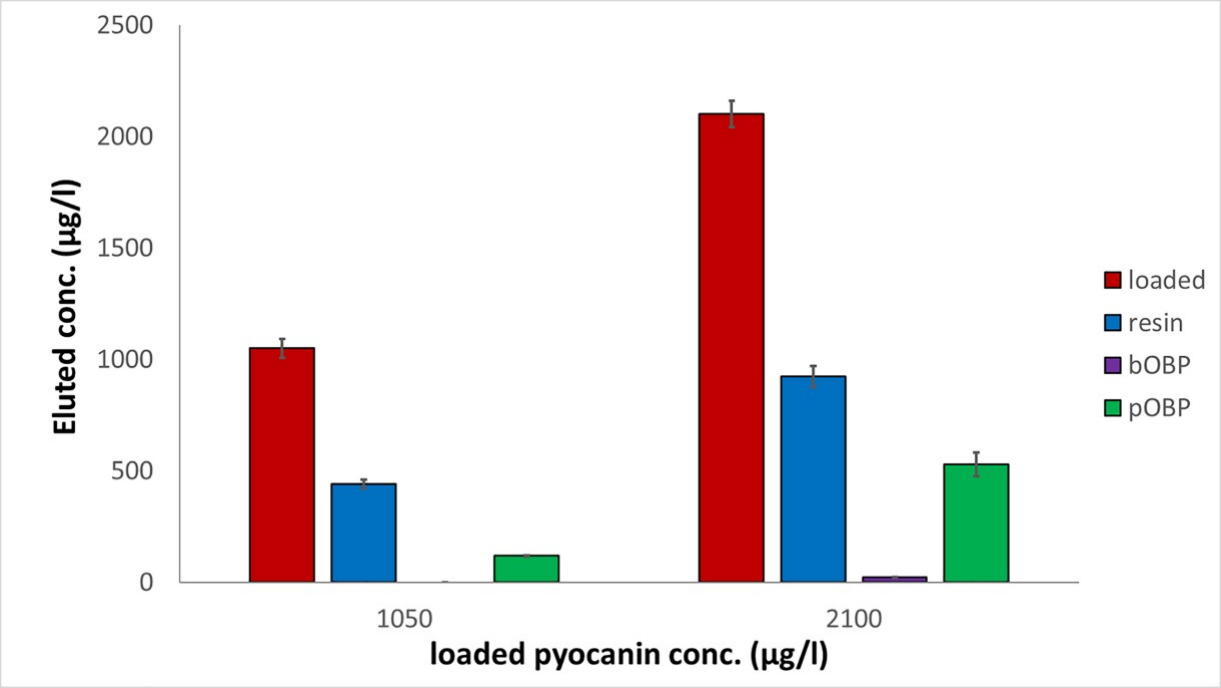
Pyocyanin removal capability of the 6-His-OBPs. Removal capability of the 6-His-bOBP and 6-His-pOBP filtering devices and of the Ni-NTA agarose resin used as control after loading aqueous solutions of pyocyanin at 1050 and 2100 μg/l, n = 3 (5 and 10 μM). (The original data of the graphs are reported in S1 Available data).

In the case of pyocyanin the results, obtained after loading solutions at 1050 and 2100 μg/l (5 and 10 μM) respectively, are depicted in Fig 2.

One way analysis of variance (ANOVA) showed the presence of significant differences among the mean values of the investigated groups (p<0.05). In order to provide detailed information on the observed differences, the post hoc Bonferroni test was also applied. By performing multiple comparisons, the presence of significant differences among the mean values of all the investigated groups was assessed (p<0.05) (S2 Table). As displayed in Fig 2, the uncoupled resin was able to retain about 50% (44±8% and 54.3±5.2% for the highest and the lowest concentration levels, respectively) of pyocyanin. The functionalization with pOBP improved the retention capabilities to 66±13% and 85±15%, whereas the linkage of bOBP allowed an almost complete binding of pyocyanin. Based on these findings, bOBP proved to be characterized by a better binding capability towards pyocyanin with respect to pOBP that, nevertheless, could retain part of the loaded ligand.

**Fig 3:**
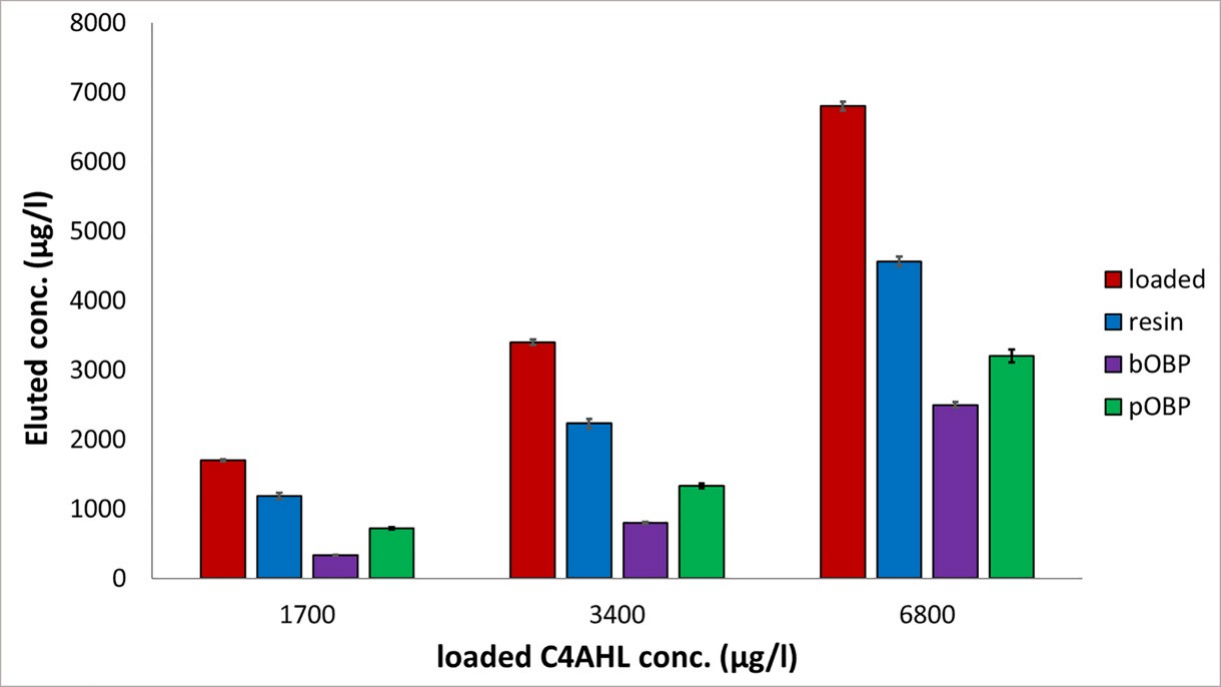
C4AHL removal capability of the 6-His-OBPs. Removal capability of the 6-His-bOBP and 6-His-pOBP filtering devices and of the Ni-NTA agarose resin used as control after loading aqueous solutions of C4AHL at 1700, 3400 and 6800 μg/l, n = 3 (10, 20 and 40 μM). (The original data of the graphs are reported in S1 Available data).

**Fig 4:**
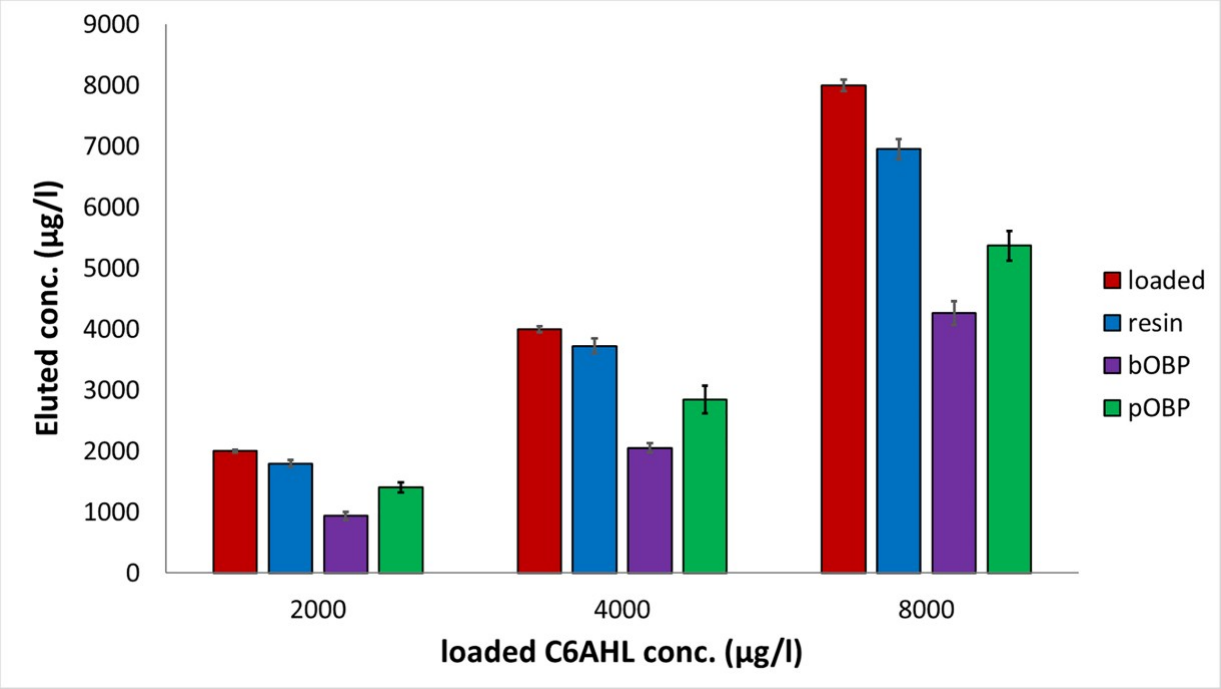
C6AHL removal capability of the 6-His-OBPs. Removal capability of the 6-His-bOBP and 6-His-pOBP filtering devices and of the Ni-NTA agarose resin used as control after loading aqueous solutions d) of C6AHL at 2000, 4000 and 8000 μg/l, n = 3 (10, 20 and 40 μM). (The original data of the graphs are reported in S1 Available data).

**Fig 5:**
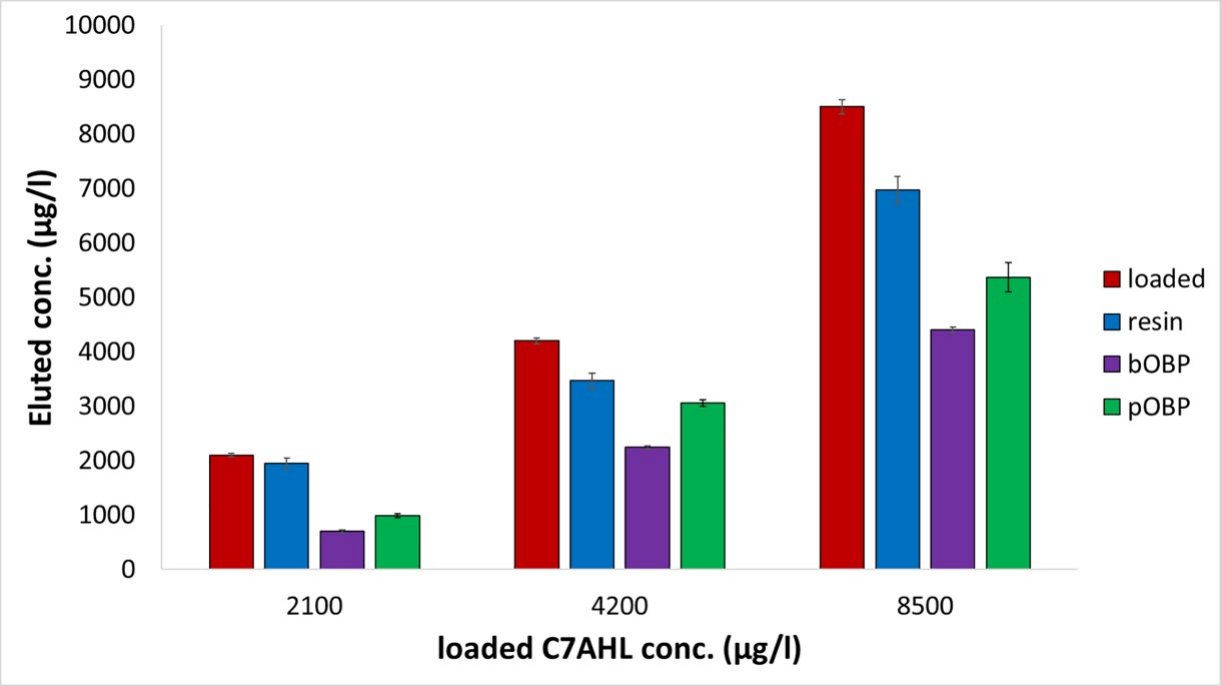
C7AHL removal capability of the 6-His-OBPs. Removal capability of the 6-His-bOBP and 6-His-pOBP filtering devices and of the Ni-NTA agarose resin used as control after loading aqueous solutions of C7AHL at 2100, 4200 and 8500 μg/l, n = 3 (10, 20 and 40 μM). (The original data of the graphs are reported in S1 Available data).

A similar behavior was observed for the C4, C6 and C7AHL (Figs 3–5). After loading solutions in the 1700–8500 μg/l range (10–40 μM), the 6-His-bOBP-agarose filter proved to be able to bind C4AHL in the 63.2±5.8%-80.6±4.2% range, C6AHL in the 47.0±10.0%-53.0±7.0% range and C7AHL in the 47.0±2.2–67.8±4.0% range, being C4AHL the most retained compound. Similarly to pyocyanin, when the 6-His-pOBP–agarose filter was used, the three AHLs were less retained by the system, being the average retentions of 41.0±9.3% (C7AHL), 30.0±2.0% (C6AHL) and 57.0±4.0% (C4AHL). Finally, the unfunctionalized resin was characterized by average retention values of 32.5±7.3%, 10.0±8.2% and 14.2±6.9% for C4AHL, C6AHL and C7AHL, respectively.

Again, ANOVA showed the presence of significant differences among the mean values (p<0.05). These findings were also confirmed by applying the post hoc multiple comparison Bonferroni test (S2 Table), thus allowing to assess the presence of significant differences among all the means of the investigated groups (p<0.05).

**Fig 6:**
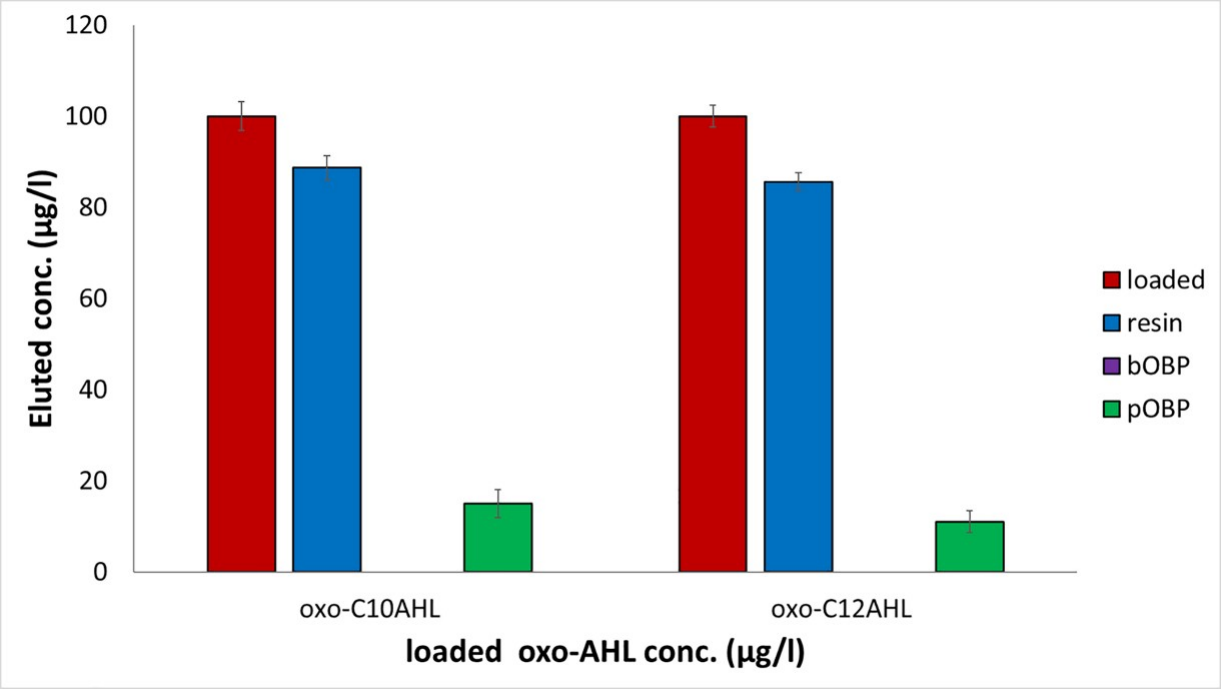
Oxo-AHLs removal capability of the 6-His-OBPs. Removal capability of the 6-His-bOBP and 6-His-pOBP filtering devices and of the Ni-NTA agarose resin used as control after loading 100 μg/l aqueous solutions of oxo-C10AHL (0.37 μM) and oxo-C12AHL (0.34 μM), n = 3. (The original data of the graphs are reported in S1 Available data).

A similar behavior was observed for both the oxo-C10AHL (0.37 μM) and the oxo-C12AHL (0.34 μM): bOBP was able to completely retain the investigated analytes, whereas pOBP was able to bind 89.2±2.4% and 85.6±3.1% of oxo-C12AHL and oxo-C10AHL, respectively (Fig 6). As reported in S2 Table, similarly to the results achieved in the case of AHLs, significant differences among the mean values were observed by applying ANOVA and Bonferroni test on the results achieved by using the oxo-compounds (p<0.05).

### Vertebrate OBPs exhibit antimicrobial activity against CA, PA and several other bacterial and yeast strains

#### Time kill assays for *Candida albicans*

**Fig 7:**
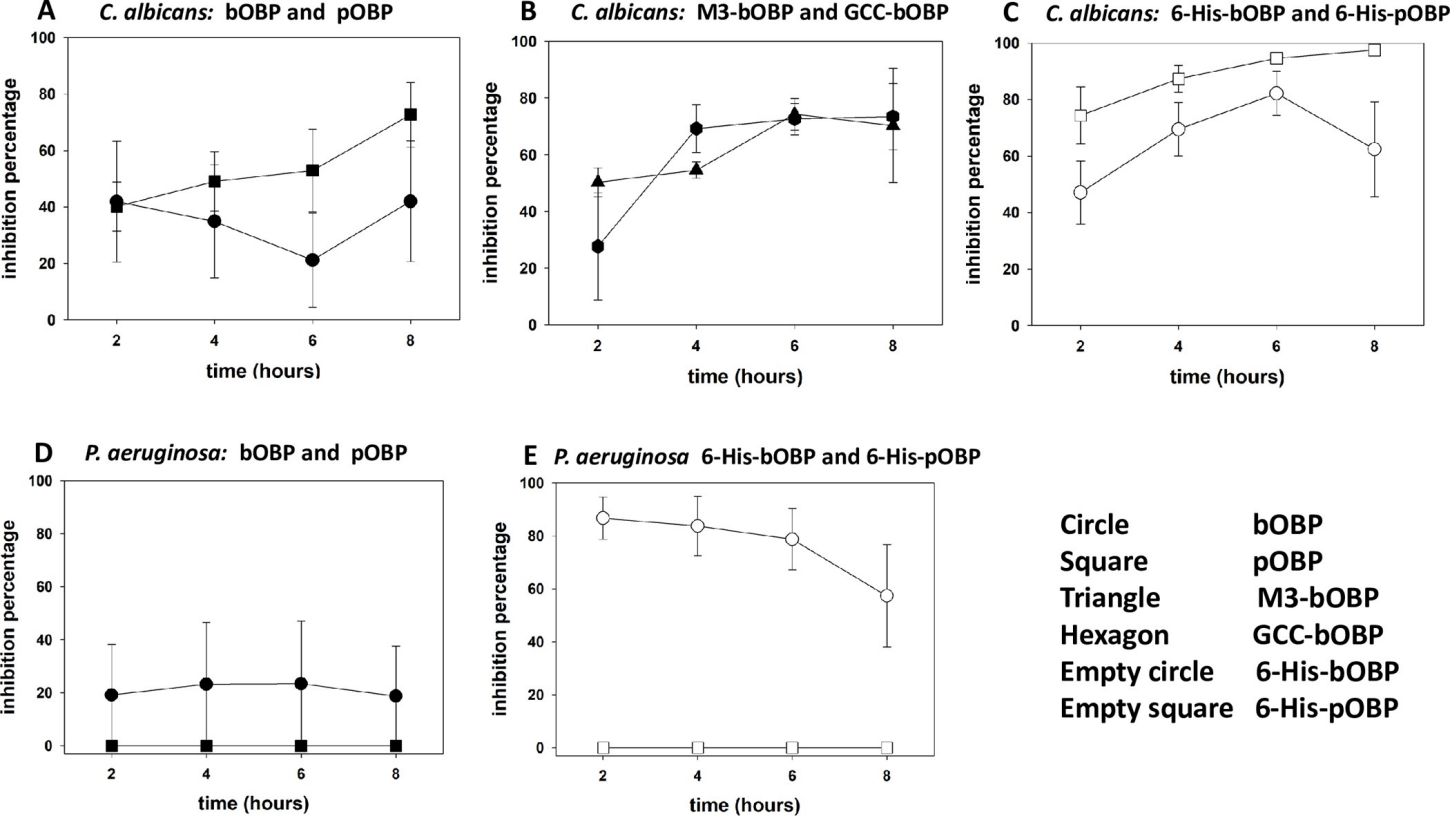
Antimicrobial activity of OBPs against *Candida albicans* and *Pseudomonas aeruginosa*. Antimicrobial activity of bOBP (circle), pOBP (square), bOBP-M3 (triangle), GCC-bOBP (hexagon), 6-His-bOBP (empty circle) and 6-His-pOBP (empty square), n = 3. The points in all the graphs are the mean values of the different replicates, while the bar is the standard error. (The original data of the graphs are reported in S1 Available data).

As reported in Fig 7A, time kill assays against CA showed that both pOBP and bOBP were able to interfere with yeast growth starting from the first two hours of contact, with about 40±21% of growth inhibition. More precisely, in the case of bOBP the TKA value was characterized by a decrease of about 20±18% after 6 hours, slightly increasing for the two remaining hours of the test. By applying the non parametric Kruskal-Wallis test, no significant differences among all the different times could be observed, thus indicating that the inhibition of the growth doesn’t significantly change for the duration of the experiment. By contrast, when pOBP was tested, a trend of the growth inhibition to a final value of about 80±8% was obtained, however, due to the high variability of the results, no significant difference could be observed among the different measurements. Since the complete inhibition could not be achieved by any of the OBP forms, it can be assumed that both proteins behave as fungistatic agents at the tested concentrations. The specificity of the antimicrobial activity of the OBP was confirmed by the negativity of the TKAs realized with the bovine form of beta-lactoglobulin as a control, that is Lipocalin with a sequence similar to those of bovine and porcine OBP (not shown).

TKAs were also performed with the two monomeric deswapped mutants M3-bOBP and CGC-bOBP (Fig 7B), thus showing their ability to inhibit the growth of CA: as shown in the figure, after 4 and 6 hours the inhibition was even higher than that obtained with pOBP. The non-paramtetric Kruskal-Wallis test revealed the presence of significant differences among the groups: as shown in the figure significant differences are observed between the inhibition growths obtained after 2 and 4 hours of contact for GCC-bOPB, and after 4 and 6 hours of contact for the M3-bOBP. Finally, Fig 7C shows that the six-histidine tag at the amino terminal is able to increase the antimicrobial activity of both the OBP forms against CA. Similarly to the results achieved by using the untagged OBP forms, the most effective protein resulted to be the 6-His-pOBP. In this case, the inhibition of the growth was 74±10% after 2 hours of contact and 97±2% after 8 hours. As for the 6-His-bOBP, the inhibitory activity started from 47±11% at 2 hours, reached the maximum value of 83±6% after 6 hours and decreased to 63±15% after 8 hours. A significant difference was observed only between 2 and 6 hours of contact.

#### Time kill assays for *Pseudomonas aeruginosa* ATCC 27853

Despite the high variability in the replicates, TKA tests showed that bOBP is able to inhibit by 20±19% the growth of PA for the eight hours of the assay (Fig 7D) with no significant differences among the inhibition growths along the TKA time. Neither pOBP nor the two monomeric mutants M3-bOBP and CGC-bOBP produced bacteriostatic activity. The same results were achieved by using bovine beta-lactoglobulin as a control, having this Lipocalin a sequence similar to those of bovine and porcine OBPs (not shown). As for the effects observed with the 6-His-bOBP (Fig 7E), after two hours of contact more than 80±8% inhibition was achieved, followed by a progressive trend to the final value of 57±19% after 8 hours, with no significant difference along the time. Finally, it can be observed that the lack of antimicrobial activity of 6-His-pOBP against PA is in agreement with the data obtained for the correspondent untagged form.

#### Other TKA assays for bacteria and Yeasts

**Fig 8:**
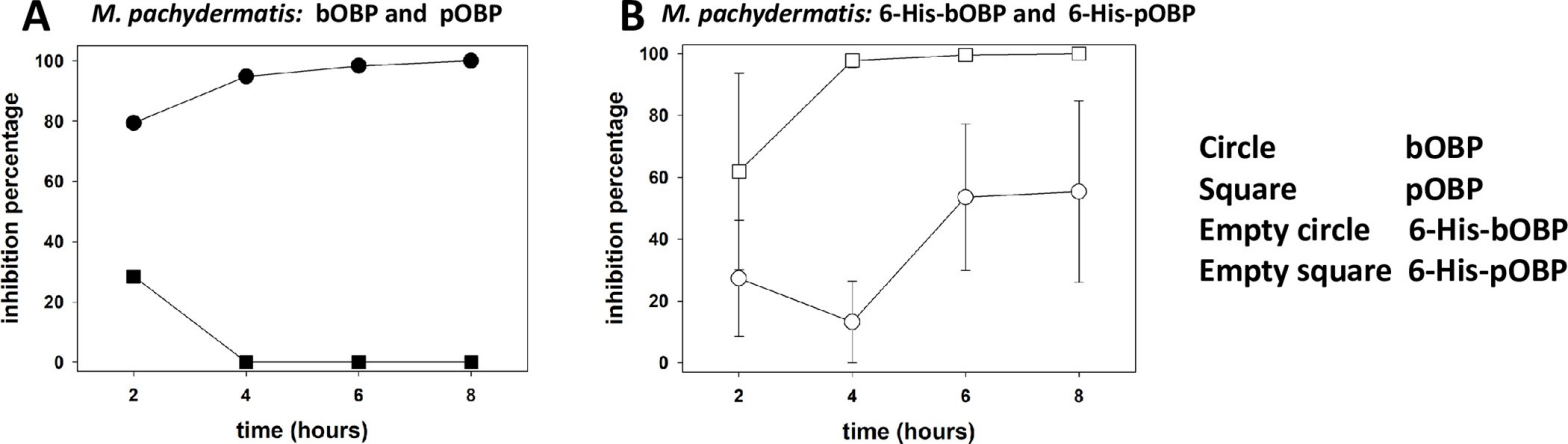
Antimicrobial activity of OBPs against *Malassezia pachydermatis*. Antimicrobial activity of bOBP (circle) n = 1, pOBP n = 1 (square), 6-His-bOBP n = 2 (empty circle) and 6XHis-pOBP n = 2 (empty square). The points of graph B are the mean values of the different replicates, while the bar is the standard error. (The original data of the graphs are reported in S1 Available data).

To assess whether vertebrate OBPs can exert antimycotic activity for pathogenic yeasts other than CA, untagged and tagged forms of both proteins were tested by TKAs against *Candida glabrata* DSM 11226 (CG) and *Malassezia pachydermatis* DSM 6172 (MP). Even though no activity was observed against CG (not shown), both proteins were able to inhibit the growth of MP (Fig 8).

However, relevant differences in terms of efficacy and time of response were observed: pOBP was characterized by a 30% (single experiment) inhibition only after 2 hours of contact, whereas the bovine form was effective for all the duration of the assays, with inhibition values progressively increasing from 80% to 100% (single experiment) between 2 and 8 hours of contact (Fig 8A). The growth inhibition curves of the tagged forms (Fig 8B) showed different behaviours from those obtained with CA (Fig 7C). The 6-His-pOBP was characterized by an evident fungistatic effect and its inhibition curve was superimposable to that obtained with the untagged bOBP. As for the efficacy of the 6-His-bOBP, the results achieved unexpectedly indicated that the presence of the six histidines determines a decrease of the efficacy with respect to untagged form. In all the experiments the statistical analysis didn’t allow to assess significant differences along the time for both protein forms.

**Fig 9:**
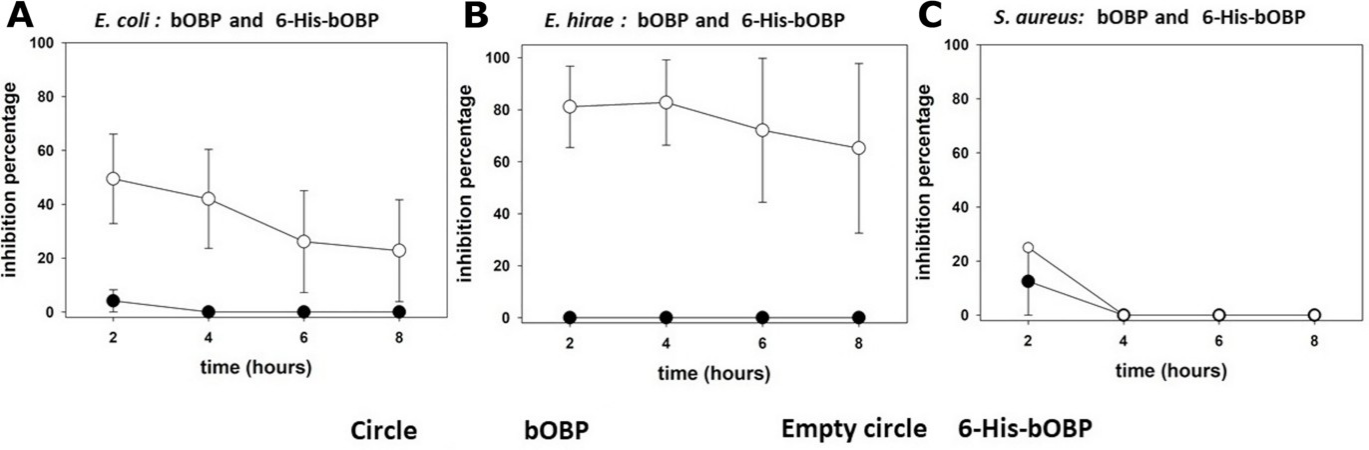
Antimicrobial activity of OBPs against *E*. *coli*, *Enterococcus hirae* and *Staphylococcus aureus*. Antimicrobial activity of bOBP (circle) and 6-His-bOBPs (empty circle), n = 2. The points in all the graphs are the mean values of the different replicates, while the bar is the standard error. (The original data of the graphs are reported in S1 Available data).

In order to verify whether the OBPs can exert direct antimicrobial activity against bacteria other than PA, the untagged and the histidine tagged forms of both proteins were tested, by TKAs, against *Enterococcus hirae ATCC10541*, *Escherichia coli* ATCC 25922, *Staphylococcus aureus* ATCC 25923 and MRSA ATCC 43300. As shown in Fig 9, a minimal antibacterial activity against *Escherichia coli* and *Staphylococcus aureus* was detected exclusively in the case of bOBP, after 2 hours of contact.

As for the tagged forms, similarly to PA, the presence of the tag increased the antibacterial activity of bOBP that could be detected, with relevant inhibition values over the entire 8 hours of the experiment for both *Escherichia coli* (Fig 9A) and *Enterococcus hirae* (Fig 9B). By contrast, the antimicrobial activity against *S*. *aureus* could be assessed only after 2 hours of contact and was slightly increased by the 6-His-bOBP (Fig 9C). Finally, no effects were observed with both untagged and tagged bOBP forms against MRSA (not shown). As already determined for PA, the lack of inhibition observed with the his-tagged form of porcine OBP confirmed its inability to exert antibacterial activity *in vitro* under the experimental conditions adopted in this study.

## Discussion

In the present study we investigated whether vertebrate OBPs might exhibit antimicrobial activity against *Candida albicans*, *Pseudomonas aeruginosa* and other pathogenic microorganisms.

The research, which was focused on the bovine and porcine forms of OBP, was divided in two steps: i) characterization of the binding capabilities of OBPs toward small diffusible molecules produced by CA and PA, i.e. farnesol, AHLs and pyocyanin, able to influence their pathogenicity as well as their ecological relationships; ii) time kill assays to highlight the direct potential antimicrobial activities of the protein against different pathogenic yeasts and bacteria.

The binding tests for the AHLs and pyocyanin were realized by incubating the ligands with submillimolar levels of OBP. This condition, on one side allows to detect the interactions between the OBPs and compounds whose affinities are lower than those of the typical ligands of the protein, and on the other it simulates the physiological environment of the mucus layering the epithelia of the respiratory tract, where the OBP is dissolved at millimolar levels. Only for the farnesol binding tests, micromolar concentrations of protein were used, proving that the OBPs dissolved in the mucus can be effective in removing quantitatively the ligand even at concentration levels closed to its limit of solubility in aqueous environment (1 μM, data sheet of the supplier). In the literature it is reported that farnesol in vitro can modulate quorum sensing in CA when it is dissolved at concentration levels close to its solubility in water [11], while data on the concentrations that trigger its biological activity in vivo have not yet been clearly established. On this basis, the biological activity of farnesol secreted by CA could be markedly influenced by a putative QQ mechanism driven by OBPs.

The OBP-based filtering devices used for the binding tests of acylhomoserine lactones, proved that the OBP forms were able to bind from 10.0±8.2% to 100% of the total quantities of the compounds dissolved in the loaded solutions. Interestingly, the highest retention capacity was determined for oxo-C12AHL, which is the QSM that regulates the LasI/Las-R QS, and that tunes the activities of RhlI/RhlR and PQS.

Therefore, the binding of 100% and 89.2±2.4% of a 0.34 μM solution of oxo-C12AHL operated by bovine and porcine OBP, respectively, suggests that an OBP-based scavenger QQ can be effective for the removal of relevant amounts of this QSM *in-vivo*, when its concentration levels are sub-micromolar. The binding tests proved lower affinities for the other AHLs tested, which could be removed from 10 to 40 μM solutions in the 30.0±2.0–80.6±4.2% range. The data indicate that an OBP-based QQ for these AHLs would be less efficient than that hypothesized for farnesol and for the 3-oxo-AHLs. Although a molecular recognition mechanism characterized by low affinity in biology is generally considered a disadvantage, a scavenging-based QQ operated by high concentration of OBPs would equally allow the host organism to operate an efficient removal of the AHLs, exclusively when they reach potentially dangerous concentration values like those that activate the QSs.

Literature data related to PA reveal that the QS driven metabolic activities, connected both to its virulence and to the competition with CA and *S*. *aureus*, are triggered by AHL concentrations ranging between 30 and 200 μM. In addition, pro-inflammatory and pro-apoptotic effects can be exerted by higher concentration levels of these QSMs [9]. Moreover several studies indicate that in the sputum of patients affected by cystic fibrosis, the levels of AHLs range between 10 and 50 nm [35, 36 and 37], while oxo-C12AHL can reach levels even higher than 1000 nM [38]. Taking into account that these measurements are not referred to the much higher concentration levels that are actually needed to trigger QS activation locally [9], the results achieved by the binding assays suggest that the OBPs, especially in the case of oxo-C12AHL, might operate QQ by scavenging significative amounts of QSMs *in vivo* from the mucus of the respiratory tract, where the proteins are dissolved at millimolar level.

The antimicrobial activity of OBPs could also be exerted by scavenging other compounds produced by pathogenic microorganisms, with physico-chemical properties and/or molecular masses comparable to those of the ligands of these proteins. As an example, the binding tests for pyocyanin showed that it can be significantly scavenged by submillimolar levels of bovine and porcine OBPs at concentration levels higher than 5 μM, being this concentration value comparable to those active *in vivo* during PA infections [39, 40 and 41]. The binding capacity of OBPs against pyocyanin is an additional indication of the potential scavenging-based antimicrobial activity of the protein against PA. Further studies dealing with other bacterial and yeast toxins, as well as with diffusible bioactive compounds having molecular characteristics comparable to those of the OBP ligands, could corroborate the hypothesis that OBPs might exert an *in vivo* scavenging-based antimicrobial activity against a wide spectrum of pathogenic microorganisms.

TKAs of both bovine and porcine OBPs against CA showed that the protein could inhibit, at submillimolar concentrations, the yeast growth with a fungistatic effect. Although bOBP was less effective than pOBP, both protein forms could inhibit the growth of CA by 40% already after 2 hours of contact. In addition, the results achieved by the TKAs with the two deswapped monomeric mutants of bOBP suggest that the typical monomeric folding of Lipocalins (present in CGC-bOBP) and/or quaternary structure of dimeric bOBP is not required for the fungistatic activity of the protein. In fact, despite the lack of the Lipocalin folding (in M3-bOBP mutant the alpha-helix is freely movable in the solvent), the presence of a functional folded beta-barrel seems to be sufficient to exert a fungistatic effect of OBP against CA. This behavior could be ascribed to the capabilities of the beta barrel alone of scavenging small molecules demanded for the proliferation of the yeast. This hypothesis was further supported by the lack of fungistatic activity against CA of bovine beta-lactoglobulin, which is a Lipocalin similar to the OBPs in terms of sequence (21% and 20% sequence homology with bovine and porcine OBP, respectively), but characterized by a more restricted ligand binding specificity [42]. In addition, the increased effect of these mutants lets suppose that the presence of a monomeric state confers to OBPs a higher fungistatic effect, regardless which amino acid residues are exposed on the surface of the protein.

Nevertheless, the experimental data obtained in the present study do not allow to establish which bioactive molecules essential for the growth of CA can be eventually scavenged by the OBPs. One candidate might be the QSM farnesol but, due to the broad ligand binding specificity of the proteins, it can be hypothesized that the scavenging may involve other molecules essential for the growth of CA.

TKAs against PA showed that only bOBP could exert direct antimicrobial activity. The bacteriostatic activity against PA was also less effective than the fungistatic effect exerted by both proteins against CA. The monomeric deswapped mutants of bOBP were not effective as antibacterial agents, thus allowing to hypothesize that the quaternary structure of the protein could be required to provide a bacteriostatic activity against PA. This behavior suggests that the antimicrobial activity against PA is a complex process involving additional mechanisms other than the broad ligand binding capacity that seems to be relevant for the fungistatic effect against CA. TKAs with OBPs from different animal species might indicate if the evolutionary pathway, which led to the protein dimerization, could be really related to the acquisition of the antibacterial activity by bovine OBP.

The higher inhibition of the growth of CA revealed by the TKAs with the amino terminal histidine tagged forms of both bovine and porcine-OBP suggests that the scavenging of metals operated by the tag might increase the antimicrobial activity of the protein. More precisely, this phenomenon is supposed to be the basis of the antifungal activity against CA operated by histatins, which are histidine-rich peptides abundantly secreted into saliva. Histatins, in fact, operate their antimicrobial activity behaving as humoral component of innate immunity by removing Cu and other metals from the surface of the yeast through the chelating properties of adjacent histidine residues [34, 43]. A similar activity could be realized by the six-histidine residues at the amino terminal of the his-tagged OBP forms, which are responsible for the interaction between the protein and the Ni ions bound to the resin in the affinity chromatography purification step. However, the absence of antimicrobial activity against PA by 6-His-pOBP strengthens the assumption that the quaternary structure of the protein might represent a discriminating element to exert antibacterial activity.

Finally, the growth inhibition curves obtained with both native and tagged bOBPs, further confirms that this protein could potentially exert direct antimicrobial activity against the yeast *Malassezia pachydermatis*, bacteria like *E*. *coli*, *S*. *aureus*, *Enterococcus hirae* and, potentially, other microorganisms present in the tissues where the protein is expressed.

As for the presence of OBPs in vertebrates, it has to be taken into account that these proteins are synthesized in all the tracts of the respiratory apparatus and then are secreted at millimolar levels into the mucus layering the epithelium. Later, the OBPs, in consequence of the unidirectional flow of the mucus driven by the coordinated beating of the cilia that cover the epithelium, reach the pharynx and pass to the digestive tract, where they are eliminated carrying the bounded ligands. Considering that in the mucus could be dissolved farnesol, AHLs and pyocyanin, and that the physiological permanence of several hours within the mucus is compatible with the time profiles of both ligand binding tests and TKAs, the experimental data obtained in this study allow to address vertebrate OBPs as humoral components of the mucus, able to contribute, by scavenging or by other mechanisms that need to be specifically investigated, to the physiological protection of the respiratory apparatus against the proliferation of PA and CA.

The broad binding specificity for AHLs and the TKAs results against other bacteria and fungi, allow also to hypothesize that vertebrate OBPs might exert antimicrobial activity against a wide spectrum of microorganisms. Further experiments and ligand binding tests for other QSMs and toxins with OBPs from different animal species, including humans, will allow to assess the validity of this assumption. In addition, vertebrate OBPs might be investigated as possible antimicrobial not-antibiotic agents for the treatment of numerous infection diseases, including those caused by PA and/or CA, in immunocompromised patients and in patients affected by cystic fibrosis.

## Supporting information

S1 Table Validation parameters for AHLs and pyocyanin binding tests. (DOCM)

S2 Table Bonferroni test results for the removal capability of the Ni-NTA agarose resin and of the 6-His-OBPs towards Pyo, C4, C6, C7AHL, oxo-C10 and oxo-C12AHL. (DOCM)

S1 Material and Methods (DOCM)

S1 Available Data (XLSX)
