# Supplementary material for: Vertebrate odorant binding proteins as antimicrobial humoral components of innate immunity for pathogenic microorganisms
Source: PLoS One. 2019 Mar 22;14(3):e0213545. doi: 10.1371/journal.pone.0213545 (PMC6430387; doi:10.1371/journal.pone.0213545)
Supplement: S1 Table — (DOCM) [file pone.0213545.s001.docm]

**S1 Table Validation parameters for AHLs and pyocyanin binding tests**

| **Analytes** | **LLOQ** | **Calibration curve** | **Within-run precision** | **Between-run precision** | **Accuracy** |
| --- | --- | --- | --- | --- | --- |
|  | **(µg/l)** |  | **(CV%)** | **(CV%)** | **(RR%)** |
| C4AHL | 39 | y=1.0863 (±0.0098)x | < 4.8 | < 4.1 | 94.81(±0.05) |
| C6AHL | 32 | y= 1.0644 (±0.0770)x | < 1.5 | < 8.2 | 96.76(±0.05) |
| C7AHL | 80 | y= 2.1435 (±0.0186)x | < 3.3 | < 14.2 | 98.08(±0.01) |
| oxo-C10AHL | 5 | y= 438.61 (±4.95)x | < 4.2 | <9.7 | 97.89((±0.03) |
| oxo-C12AHL | 5 | y= 328.68 (±3.61)x | < 5.1 | < 7.2 | 96.68(±0.05) |
| Pyocyanin | 82 | y= 0.0105 (±0.0003)x | < 16.0 | < 15.2 | 92.62(±0.03) |
