# Supplementary material for: Vertebrate odorant binding proteins as antimicrobial humoral components of innate immunity for pathogenic microorganisms
Source: PLoS One. 2019 Mar 22;14(3):e0213545. doi: 10.1371/journal.pone.0213545 (PMC6430387; doi:10.1371/journal.pone.0213545)
Supplement: S2 Table — (DOCM) [file pone.0213545.s002.docm]

**S2 Table**

Bonferroni test results for the removal capability of the Ni-NTA agarose resin and of the 6-His-OBPs towards Pyo, C4, C6, C7AHL, oxo-C10 and oxo-C12AHL (n=3).

| **PYO (µg/l) mean values ± standard deviation** | | | |
| --- | --- | --- | --- |
| nominal | **1050** | **2100** |  |
| loaded | 1055±71^a^ | 2096±106^e^ |  |
| resin | 480±22^b^ | 956±63^f^ |  |
| bOBP | n.d. | 23±2 ^g^ |  |
| pOBP | 157±10^d^ | 615±66 ^h^ |  |
| **C4AHL (µg/l) mean values ± standard deviation** | | | |
| nominal | **1700** | **3400** | **6800** |
| loaded | 1690±46^aI^ | 3400±79^eI^ | 6780±85^iI^ |
| resin | 1186±83^bI^ | 2231±160^fI^ | 4564±351^lI^ |
| bOBP | 330±14^cI^ | 800±46^gI^ | 2507±145^mI^ |
| pOBP | 720±25^dI^ | 1330±56^hI^ | 3200±142^nI^ |
| **C6AHL (µg/l) mean values ± standard deviation** | | | |
| nominal | **2000** | **4000** | **8000** |
| loaded | 2011±33^aII^ | 4020±72^eII^ | 8049±84^iII^ |
| resin | 1852±146^bII^ | 3618±312^fII^ | 6953±572^lII^ |
| bOBP | 939±68^cII^ | 2054±180^gII^ | 4235±424^mII^ |
| pOBP | 1411±26^dII^ | 2886±62^hII^ | 5368±113^nII^ |
| **C7AHL (µg/l) mean values ± standard deviation** | | | |
| nominal | **2100** | **4200** | **8500** |
| loaded | 2093 ± 31^aIII^ | 4180±56^eIII^ | 8510±130^iIII^ |
| resin | 1950 ±132^bIII^ | 3465 ± 235^fIII^ | 6970±488^lIII^ |
| bOBP | 677 ± 27^cIII^ | 2249 ± 54^gIII^ | 4501±97^mIII^ |
| pOBP | 990 ±82^dIII^ | 2858 ± 258^hIII^ | 5361±574^nIII^ |
| **C10oxo (µg/l) mean values ± standard deviation** | | | |
| nominal | **100** |  |  |
| loaded | 102±8^aIV^ |  |  |
| resin | 89±4^bIV^ |  |  |
| bOBP | n.d. |  |  |
| pOBP | 15±3^cIV^ |  |  |
| **C12oxo (µg/l) mean values ± standard deviation** | | | |
| nominal | **100** |  |  |
| loaded | 99±9^aV^ |  |  |
| resin | 86±6^bV^ |  |  |
| bOBP | n.d. |  |  |
| pOBP | 11±2^cV^ |  |  |

-: not detected

Different letters on the mean values denote significant differences (p<0.05).

Data with the same letters are not significantly different.
