# Supplementary material for: Vertebrate odorant binding proteins as antimicrobial humoral components of innate immunity for pathogenic microorganisms
Source: PLoS One. 2019 Mar 22;14(3):e0213545. doi: 10.1371/journal.pone.0213545 (PMC6430387; doi:10.1371/journal.pone.0213545)
Supplement: S1 Material and Methods — (DOCM) [file pone.0213545.s003.docm]

**S1 Materials and Methods**

**Reagents**

N-butanoyl-L-homoserine lactone (≥ 95% purity, C4AHL), N-hexanoyl-L-homoserine lactone (≥98% purity, C6AHL), N-heptanoyl-L-omoserine lactone (≥97% purity, C7AHL), N-(3-oxodecanoyl)-L-homoserine lactone (oxo-C10AHL), N-(3-oxodedecanoyl)-L-homoserine lactone (oxo-C12AHL), pyocyanin from *Pseudomonas aeruginosa* (≥98% purity), potassium phosphate dibasic trihydrate (≥99% purity), potassium dihydrogen phosphate (≥99% purity), dimethylformamide (≥99.8% purity, DMF) and 1-aminoanthracene (≥90% purity, AMA) and bovine Lactoglobulin were from Merck (Milan, Italy).

**bOBP and pOBP biosynthesis**

Untagged and histidine tagged Bovine and porcine OBP, and the two unswapped mutants of bOBP were purified as previously reported [1-3]. To avoid the presence of traces of antimicrobial substances in the purified protein that might have affected the time kill assays (TKA) tests against the different microorganisms, the culture media did not contain ampicillin and the bacterial cell disruption procedure was realized in the absence of Lysozyme.

Functionality of the protein in solution was determined by direct titrations using the fluorescent ligand 1-aminoanthracene (AMA), as previously reported [1-3].

**Binding tests for 1-aminoanthracene and farnesol**

Functionality of the different OBP forms was evaluated by direct titrations using the fluorescent ligand 1-aminoanthracene (AMA) [1 and 2] that was prepared as a stock solution at a concentration of 20 mM in ethanol without additives. Briefly, 1 ml samples of OBP (0.5 µM for bOBP and 1 µM for the other forms), in 20 mM tris-HCl buffer pH 7.8, were incubated for 30 minutes at room temperature in the presence of increasing concentrations of AMA (0.156 - 10 µM). Fluorescence emission spectra between 450 and 550 nm were recorded with a Perkin-Elmer LS 50 luminescence spectrometer (excitation and emission slits of 5 nm) at a fixed excitation wavelength of 380 nm and the formation of the AMA-OBP complex was followed as an increase of the fluorescence emission intensity at 480 nm. The dissociation constants of the AMA-OBP complexes were determined from the hyperbolic titration curves using the nonlinear fitting program of Sigma Plot 5.0 (Cambridge Soft. Corp., Cambridge, MA, USA).

The dissociation constants of complexes between the Odorant binding-protein tagged at the amino terminal forms and farnesol were determined by competitive binding tests with the fluorescent ligand 1-aminoanthracene, as previously described for other ligands [4, 5]. Each OBP (0.5 µM for bOBP and 1 µM pOBP) was dissolved in 20 mM Tris/HCl buffer pH 7.8 and pre-incubated at room temperature for 20 min. with 1-aminoanthracene (0.7 µM). 1 ml samples were then poured into different tubes containing increasing amounts of farnesol from 0.2 to 25.0 µM. The stock solutions of farnesol and AMA were prepared at a concentration of 20 mM in ethanol without additives. The samples were incubated for 30 min at room temperature and the binding of farnesol was assessed as a decrease of the fluorescence emission of the AMA-OBP complex at 480 nm upon excitation at 380 nm (excitation and emission slits 5 nm). As a negative control, fluorescence emission spectra between 450 and 550 nm were recorded for ‘blank solutions’ containing 0.7 µM 1-aminoanthracene and the amounts of ethanol present in the different samples of farnesol considered for the competition. The apparent K_d_ values for the binding complexes were determined from the competition curves analyzed as two parameters hyperbolic decays with the non-linear fitting function of the software ‘Sigma plot 5.0’ (Cambridge soft. Corp., Cambridge MA, USA). The true Kd values were then calculated from the following formula [6]:

$$K_{d}=K_{d}\mathrm{app}\frac{1}{1+\left( \frac{1}{K_{d}\mathrm{AMA}}\cdot\left[ \mathrm{AMA} \right] \right)}$$

where K_d_AMA is the dissociation constant of the AMA-OBP complex and K_d_app is the concentration of farnesol that displaced 50% of the AMA bound to the OBP.

**Binding tests for AHLs, 3-oxo-AHLs and pyocyanin**

**Stock and working solutions.** Stock solutions of C4AHL, C6AHL and C7HSL, oxo-C10AHL and oxo-C12AHL at 2000 mg/l were prepared in DMF and stored at -20°C. Working solutions at the proper concentration level were prepared in distilled water by dilution from the stock solutions.

The standard solution of pyocyanin was prepared in water at the concentration of 1000 mg/l and stored at -20 °C. Working solutions were prepared in a phosphate buffer (20mM, pH=7.8) by proper dilution from the stock solution.

**Binding test.** The binding tests for the AHLs, the 3-oxoAHL and pyocyanin were performed by using filtering devices having porcine and bovine OBP as retention elements. To this aim the two amino-terminal 6-his tagged proteins were coupled to Ni-NTA agarose beads, as previously described [7].

The binding capability of the 6XHis-OBPs-based filters was evaluated by loading 100 µL of 6-His-bOBP or 6-His-pOBP-agarose suspensions in 1 ml syringe and filtering 300 µl of the following aqueous solutions:

1. C4AHL at 1.7, 3.4 and 6.8 mg/l (10, 20 and 40 µM);
2. C6AHL at 2,4 and 8 mg/l (12 and 40 µM)
3. C7AHL at 2.1, 4.2 and 8.5 mg/l (10, 20 e 40 µM)
4. oxo-C12AHL at 100 µg/l (0.34 µM)
5. oxo-C10AHL at 100 µg/l (0.37 µM);
6. pyocyanin 1.0 and 2.1 mg/l (4.8 and 10 µM)

Control experiments were performed using the unfunctionalized Ni-NTA agarose resin. The eluates were then analysed by means of GC-MS for the AHLs, LC-MS/MS for the oxo-AHLs and UV-vis for and pyocyanin, respectively.

Three independent replicated measurements were always performed.

**GC-MS analysis.** The GC-MS analyses were performed on a HP 6890 Series Plus gas chromatograph (Agilent Technologies, Palo Alto, CA) equipped with a MSD 5973 mass spectrometer (Agilent Technologies). Helium was used as the carrier gas at a constant flow rate of 1.3 mL/min; the gas chromatograph operated in splitless mode for 1 min with the PTV injector (Agilent Technologies) maintained at the temperature of 250 °C and was equipped with a 1.5 mm i.d. multibaffled liner (Agilent Technologies).

Chromatographic separation was performed on a 30 m x 0.25 mm, df 0.25 mm HP-5 ms capillary column (Agilent Technologies), using the following temperature program: 100°C, 15°C/min to 250°C, 250°C for 1 min. The transfer line and source were maintained at 220 and 150°C, respectively. Preliminarily, full scan EI data were acquired to determine appropriate masses for selected-ion monitoring mode used for HLs quantitation (m/z 143, 125, 171for C4 AHL; m/z 143, 125, 156 for C6AHL; m/z 143, 43, 57 for C7AHL) under the following conditions: solvent delay: 4 min; ionization energy: 70 eV; dwell time: 30 ms; electron multiplier voltage: 2200 V. For all the investigated analytes, the most abundant ion was used for quantitation, whereas the corresponding ion ratios were used for confirmation purposes. Signal acquisition and data handling were performed using the HP Chemstation (Agilent Technologies).

**LC-MS/MS analysis**. LC-MS/MS analysis was performed on a HPLC system (Thermo Electron Corporation, San Josè, CA, USA) coupled with a LTQ XL linear ion trap mass spectrometer (Thermo Electron Corporation) equipped with a pneumatically assisted electrospray (ESI) interface. The system was controlled by the Xcalibur software (Thermo Electron Corporation). A volume of 15 µl of eluate was directly injected into a C18 Kinetex (100 mm×2.1 mm, 2.6 μm particles) (Phenomenex, CA, USA) column thermostated at 25 °C. A binary solvent gradient was used for the analysis (flow rate: 300 µl/min): solvent A consisted of 0.1 % (v/v) formic acid in water and solvent B of 0.08 % (v/v) formic acid in acetonitrile; for elution, solvent B was delivered by a linear gradient from 50% to 90% in 6 min, then it was maintained at 90 % for 1 min before column re-equilibration. Optimized conditions of the source were set as follows: positive ion mode; sheath gas (nitrogen, 99.99% purity), 30 arbitrary units; auxiliary gas (nitrogen, 99.99% purity) 10 arbitrary units; sweep gas (nitrogen, 99.99% purity), 5 arbitrary units; ESI voltage, 3.5 kV; capillary voltage, 20 V; capillary temperature, 250 °C; tube lens, 40 V. Product ion scan was used as MS/MS acquisition mode; extracted ion chromatograms were obtained by extraction of individual fragment ion currents using Xcalibur software. The monitored precursor ion/product ion transitions are the following: m/z 270/102 (for quantitation), m/z 270/169 and m/z 270/242 for oxo-C10AHL (normalized collision energy, 15); m/z 298/197 (for quantitation), m/z 298/102 and m/z 298/280 for oxo-C12AHL (normalized collision energy, 15).

**UV-vis analysis**. A UV-vis Thermo Evolution 260 Bio spectrophotometer equipped with a SPE 8w Peltier Water Cooled Cell-Changer was used for pyocyanin determination in the 300-750 λ range. A Thermo Insight software was used for data acquisition. The pyocyanin solutions were analyzed under the following conditions: virtual dual beam optics; 100% baseline correction; band width: 1 nm; integration time: 0.25 s.

**Method validation.** Method validation was performed according to the guidelines for bioanalytical method validation [8] using phosphate buffer (20 mM, pH 7.8) as blank matrix. Briefly, the lower limit of quantification (LLOQ) was calculated as signal to noise ratio, S/N = 5, using five independent samples and tested for accuracy and precision to meet the previously cited criteria. The calibration curves were evaluated on six concentration levels in the following ranges: LLOQ - 4000 μg/l for C4AHL and C6AHL; LLOQ - 8000 μg/l for C7AHL; LLOQ - 2500 μg/l for pyocyanin; LLOQ-2000 μg/l for oxo-C10AHL and oxo-C12AHL, respectively. Three replicated measurements for each level were performed. Homoschedasticity was verified by applying the Bartlett test. Mandel’s fitting test was also performed to check the goodness of fit and linearity. The significance of the intercept (significance level 5%) was established by running a Student’s t-test.

Precision in terms of both within-run and between-run precision in terms of RSD% on three concentration levels i.e. the LLOQ, the intermediate and high levels used for the evaluation of linearity, by performing five replicated measurements for each level. Between-run precision was estimated over three days verifying homoscedasticity of the data and performing the analysis of variance (ANOVA) at the confidence level of 95%.

Accuracy was calculated in terms of recovery rate (RR%) as follows:

$$RR\%=\frac{c_{1}}{c_{2}}\cdot100$$

where c_1_ is the measured concentration and c_2_ is the concentration calculated from the quantity spiked into the sample. The same concentration levels previously described were analyzed by performing five replicated measurements.

Method selectivity was assessed by testing for interference 6 blank phosphate buffer (20 mM, pH 7.8) samples. Finally, stability was evaluated in terms of both long-term and short-term (bench-top storage), and after freeze and thaw cycles. The stability of the stock solutions was evaluated by comparison to freshly made calibrators. Three replicates at the LLOQ and at the intermediate level used for each calibration curve were always performed.

**Microbiological assays**

**Bacterial and fungal strains.** Antimicrobial activities of OBPs and the bovine form of the Lipocalin beta-lactoglobulin were evaluated against bacterial and fungal reference strains: *E. coli* ATCC 25922, *Enterococcus hirae* ATCC 9790, *Staphylococcus aureus* MRSA ATCC 43300, *Pseudomonas aeruginosa* ATCC 27853, *Candida albicans* ATCC 11006, *Candida glabrata* DSM 11226 (CG) and *Malassezia pachydermatis* DSM 6172 [9].

Bacterial and fungal colonies from fresh cultures of each tested strain were inoculated in appropriate medium and incubated 24 and 48 hours, for bacteria and yeasts respectively, at 37°C in aerobic atmosphere. After incubation, suspensions were centrifuged 20 minutes at 2000 rpm and 4°C and the pellet resuspended in phosphate buffer (PB) 10mM pH 7.

Bacterial suspensions were investigate via UV-vis analysis (absorbance at 600 nm) in order to reach optical densities in the 0,08-0,13 OD range. Fungal suspensions were diluted to a concentration equal to 5 according to the McFarland turbidity scale. The reported concentrations correspond to 10^8^ CFU/ml, both for bacterial and fungal suspensions (Clinical Laboratory Standards Institute, 2008). All suspensions were further diluted 1:100 in PB to obtain a final concentration of 10^6^ CFU/ml. Within 30 minutes after preparation, bacterial and fungal suspensions were used for the microdilution assay.

**Time kill assay.** Antimicrobial activity was evaluated in function of time through time-kill assays [S10]. In a 96 U bottomed wells microtiter plate, 75 µl of OBP solution were inoculated with an equal volume of bacterial or fungal suspension, adjusted at the appropriate titer as above mentioned. Final protein concentrations were 50 µM for dimeric bOBP and 6-His-bOBP, and 50 µM for pOBP, M3-bOBP, GCG-bOBP and 6-His-pOBP. Control experiments were realized by replacing the different OBP forms with the Lipocalin beta-lactoglobulin (50 µM). Growth and sterility controls were set for each strain. The plates were then incubated at 37°C in aerobic atmosphere for the whole duration of the test. After 0, 1, 2, 4, 6, and 8 hours of contact, 20 µl of the suspension of each wells were inoculated in 2ml of an appropriate liquid medium (Mueller-Hinton or Brain-Heart-Infusion broth) and 20 µl were plated on solid agar medium (Columbia blood agar with 5% of bovine erythrocytes for bacterial strains and Sabouraud agar for yeasts). Agar plates were incubated for 24 or 48 hours for bacteria and yeasts, respectively at 37°C in aerobic atmosphere, then CFU were counted.

**References**

1. Ramoni R, Vincent F, Ashcroft AE, Accornero P, Grolli S, Valencia C, Tegoni M, Cambillau C. 2002. Control of domain swapping in bovine odorant-binding protein. Biochem J 365: 739-748.

2. Grolli S, Merli E, Conti V, Scaltriti E, Ramoni R. 2006. Odorant binding protein has the biochemical properties of a scavenger for 4-hydroxy-2-nonenal in mammalian nasal mucosa. FEBS J 273: 5131–5142.

3. Ramoni R, Spinelli S, Grolli S, Conti V, Merli E, Cambillau C, Tegoni M. 2008. Deswapping bovine odorant binding protein. Biochim Biophys Acta 1784: 651-657.

4. Vincent F, Spinelli S, Ramoni R, Grolli S, Pelosi P, Cambillau C, Tegoni M. 2000. Complexes of porcine odorant binding protein with odorant molecules belonging to different chemical classes. J Mol Biol 300: 127-139.

5. Vincent F, Ramoni R, Spinelli S, Grolli S, Tegoni M, Cambillau C. Crystal structures of bovine odorant-binding protein in complex with odorant molecules*.* Eur J Biochem 2004;271: 3832-3842.

6. Ramoni R, Vincent F, Grolli S, Conti V, Malosse C, Boyer FD, Nagnan-Le Meillour P, Spinelli S, Cambillau C, Tegoni M. 2001. The Insect Attractant 1-Octen-3-ol Is the Natural Ligand of Bovine Odorant-binding Protein. J Biol Chem 276: 7150–7155.

7. Bianchi F, Basini G, Grolli S, Conti V, Bianchi F, Grasselli F, Careri M, Ramoni R. 2013. An innovative bovine odorant binding protein-based filtering cartridge for the removal of triazine herbicides from water. Anal Bioanal Chem 405: 1067–1075.

8. Guidance for Industry, Bioanalytical Method Validation, US Department of Health and Human Services, Food and Drug Administration, 2018. https://www.fda.gov/downloads/drugs/guidances/ucm070107.Pdf , last access: June, 6 2018.

9. CLSI. Methods for Dilution Antimicrobial Susceptibility Tests for Bacteria That Grow Aerobically; Approved Standard-Tenth Edition. CLSI document M07-A10. Wayne, PA: Clinical and Laboratory Standard Institute; 2015.

S10. Balouiri M, Sadiki M, Ibnsouda SK. 2016. Methods for in vitro evaluating antimicrobial activity: A review. J Pharm Anal 6: 71-79
